# Host plants determine oviposition preference and larval fitness of tomato leafminer, *Phthorimaea absoluta*

**DOI:** 10.3389/finsc.2026.1854641

**Published:** 2026-06-04

**Authors:** Tariku Tesfaye Edosa, Kwang-Ho Kim, Sung-Wook Jeon, Jaekun Kim, Jong-Ho Park, Hyunoh Sun

**Affiliations:** Pests and Weed Control Division, Rural Development Administration, Jeonju, Republic of Korea

**Keywords:** development, growth indices, leaf damage, solanaceous plants, tomato

## Abstract

*Phthorimaea absoluta* (Meyrick 1917) (Lepidoptera: Gelechiidae) was accidentally introduced into South Korea in 2024 and is currently distributed throughout the country’s tomato-producing areas. The interaction of the insect with native alternative host plants in Korea has not been elucidated. Therefore, in the current study, we have determined the oviposition preference and larval development of *P. absoluta* in Korean native weed and crop plants (two each from Solanaceae). The evaluation of oviposition preference was conducted using a choice and a no-choice test. First-instar larvae of the tomato leafminer were placed on each tested plant and monitored daily for mortality and head capsule measurements to determine larval instars. Accordingly, female adults highly preferred tomato and black nightshade for oviposition, and the highest survival percentage was also observed from these plants. On the other hand, the larvae reared on the eggplant and devil’s apple took a longer time to pupate, and the highest mortality was recorded. The positive correlation between larval duration and larval mortality, and the negative correlation between larval duration and pupal weight were observed. The highest growth and fitness indices were observed in larvae reared on tomato and black nightshade. Collectively, tomato leafminer can complete its life cycle on all the tested plants with varying performance. Therefore, it is important to consider these plants as potential alternative hosts in predicting the insect abundance and planning the integrated pest management strategies of the pest.

## 1 Introduction

Tomato leafminer, *Phthorimaea absoluta* (Meyrick 1917) (Lepidoptera: Gelechiidea), is native to South America and presently spreads across the country, causing significant tomato infestation and yield loss (1). Currently, the insect has spread to tomato-producing regions in Europe, Africa, the Middle East, and Asia, threatening both open-field and greenhouse tomato production (2–4). The pest has a wide host range and a short life cycle that enables it to adapt to new areas. The pest is multivoltine and can produce up to 12 generations in a year under a suitable environment (3, 5–7). Even though general host ranges have been reported, the oviposition preference, larval fitness, and developmental times on such host plants have not been intensively assessed in recently infested areas.

Host preference patterns of plant-feeding insects may change following population invasions of new plant species, and this phenomenon can involve changes in adult oviposition behavior, larval feeding behavior, and development (8, 9). In addition, the host plant optimization process used by herbivores could limit larval developmental performance on other hosts (10–12). The availability of new hosts facilitates the expansion of an insect beyond the geographic scope of native hosts.

Tomato leafminer was accidentally introduced into South Korea in 2024 and has spread to tomato-growing areas in the region (13). The tomato leafminer monitoring covered nine provinces of South Korea, showing that tomato leafminer occurrence was recorded in all surveyed areas across all seasons (14). As the pest is new in the country, the interaction of the insect with native alternative host plants has not been elucidated. Understanding the ovipositional preference and larval performance on the alternative hosts will enable us to predict the abundance of the pest and forecast the insect incidence. The findings greatly contribute in developing sustainable and effective IPM measures, as this provides the biological characteristics of the pest across four alternative host plants.

## 2 Materials and methods

### 2.1 Planting materials

The experiments were conducted at the Rural Development Administration (RDA), Insect and Weed Management Laboratory, Wanju, Republic of Korea. Solanaceous crops and weed species, namely *Solanum melongena* L. 1753 (eggplant), *Solanum linnaeanum* Hepper & P. M.L. Jaeger 1986 (devil’s apple), *Solanum nigrum* L. 1753 (black nightshade), and *Solanum lycopersicum* L. 1753 (tomato), were tested as alternative host plants. The seedlings of eggplant (Heuck jinju variety), black nightshade (weed), and tomato (Tamina variety) were grown in pots filled with baroker seedling growing soil (nitrate-nitrogen (200-350, ammonium nitrate (<150mg/l), effective phosphate (200-350), minerals (potassium, calcium, magnesium, copper, iron, zinc, and boron). Devil’s apple (weed) seedlings were collected from the Wonju area, Republic of Korea, and raised in pots at a greenhouse. All the seedlings were watered daily and kept in insect-proof cages for 3–5 weeks, and it was used for the experiments at the age of 5–6 leaf stages.

### 2.2 Tomato leafminer stock colony

We obtained the tomato leafminer for the bioassay from a colony maintained in the Insect and Weed Management Laboratory, which sourced the insects originally from commercial tomato production sites in the Republic of Korea, Gyeonggi-do, Paju city, Papyeong-myeng 17-4, during 2024. The tomato leafminer population used in the bioassay study was the 20^th^ generation of the laboratory-reared population. The laboratory colony was reared on a tomato plant (Tamina variety) under controlled laboratory conditions (Temperature: 25 ± 0.5 °C, photoperiod: 12 h L: 12 h D, and relative humidity: 75 ± 1%). The larval instars were separately reared in Plexiglas cages (40 × 40 × 40 cm) covered with fine mesh netting. The insect-free pot-grown tomato plants were provided for oviposition and larval feeding.

### 2.3 Tomato leafminer oviposition preference

Oviposition preference experiments were performed in steel-framed mesh-covered cages. The experiment was conducted using a choice and a non-choice test.

#### 2.3.1 Choice test

One plant of each tested host (eggplant, devil’s apple, black nightshade, and tomato) was grown until 5–6 leaves in a plastic pot and randomly placed in a rectangular glass cage. The upper side of the cages was covered with fine mesh for ventilation. Pupae (1-day old) were collected and sexed based on morphological features (15). Newly emerged five male and five female adults were released per cage and fed with 10% honey solution.

The experiment was arranged in a randomized complete block design (RCBD) with five replicates. The number of eggs laid was recorded every other day, as female *P. absoluta* lay the majority of their eggs within a week (16).

The experiment was conducted in RCBD and replicated five times. The number of eggs laid was assessed and recorded on alternative days of the week, as the female *P. absoluta* lays the maximum eggs within a week (16). Fifty eggs from each host plant were incubated at 25 ± 2°C and 87 65 ± 5% RH to estimate the egg hatchability and leaf mining by the neonates within 24 h of hatch.

#### 2.3.2 No-choice tests

The four plant species tested for oviposition preference in the choice test were also evaluated in a no-choice test. The procedure was the same as in the choice test; however, only one plant of a test plant species was placed in each experimental cage. Similarly, five pairs of female and male of the *P. absoluta* were released into each cage. The experiment was arranged in a Randomized Complete Block Design (RCBD) with five replications.

### 2.4 Survival and development time of larval instars

Thirty larvae hatched from each evaluated plant species were separately placed on the leaf of their respective host plant. The leaves were kept in the SPL insect breeding dish (60 × 15 mm with 3.2 mm ventilation mesh and 0.53µm pores), lined with a cotton wool pad and filter paper. The filter paper was monitored daily, and it was moistened with tap water to maintain the turgidity of the leaves. Each larva was monitored daily for the head capsule to determine the larval instars. The same leaf was provided as food until adult emergence. Larval survivability, each larval period, pupation, and adult emergence were recorded. Upon pupation, the pupae were sexed (15), weighed, and placed into separate Petri dishes for each sex and plant species until adult eclosion. The pupae were weighed using a sensitive digital balance (precision ± 0.001mg).

### 2.5 Leaf damage assessment

**Figure 1:**
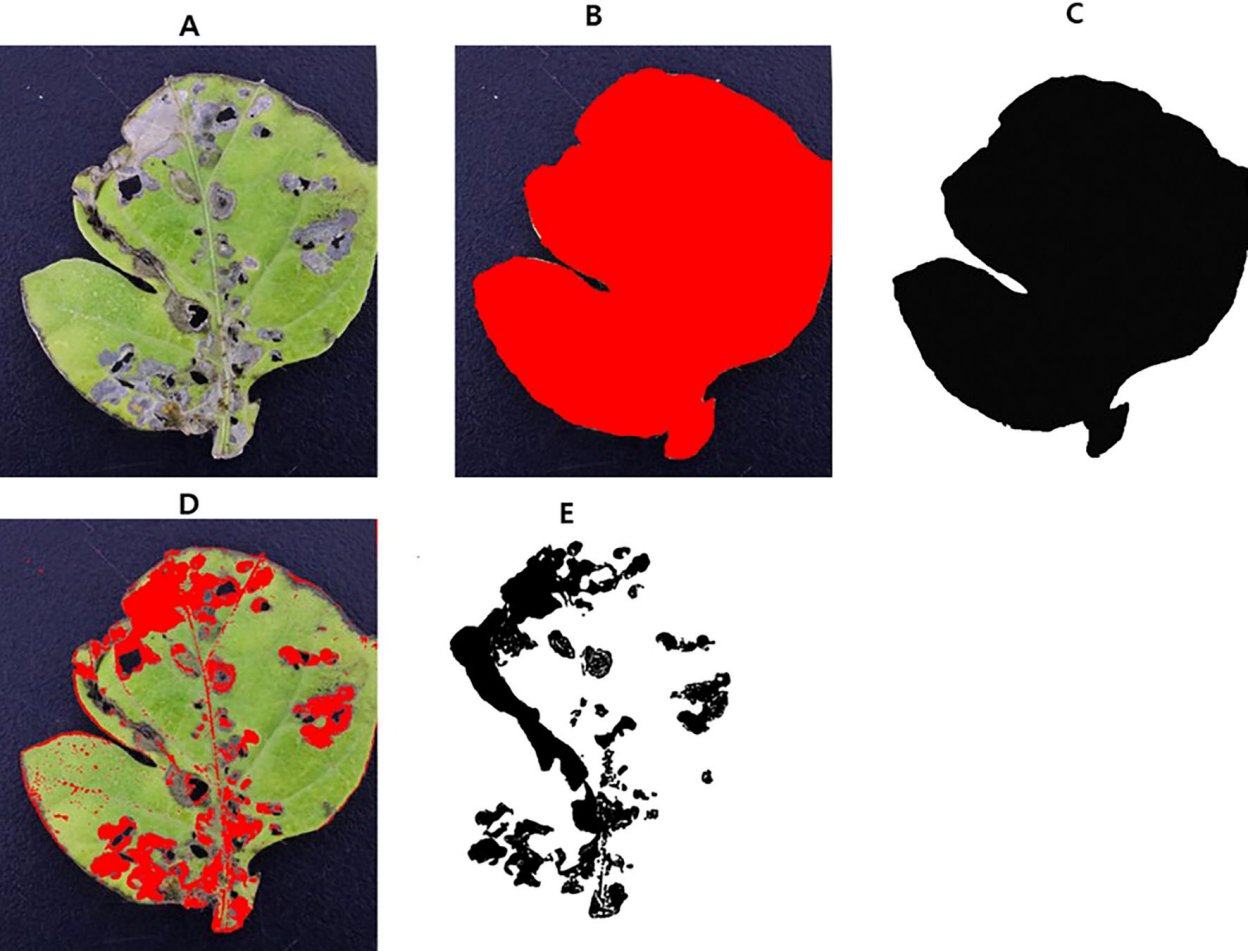
Damaged leaf image analyses using ImageJ. Illustrating the progression from **(A)** original image, **(B)** image adjust via colour threshold in ImageJ, **(C)** binary image, **(D)** damaged area separated via colour threshold **(E)** damaged area image changed to binary.

After 7^th^ and 14^th^ days of neonate larval introduction, the leaf damage per larva was measured. The picture of five damaged leaves of each plant host was captured with an ultrasonic Sony camera (Model Sony Alpha 7 (ILCE-7)). To ensure good contrast between the leaf and background, a black background was used. Image processing and quantification of leaf damage by the larvae were performed with ImageJ (v.2.0.0 with Java 1.6.0_24). Briefly, the image was opened in the ImageJ software, and the image was converted to grayscale (Go to Image > Type > 8-bit). To separate the leaf image from the background, the threshold of the leaf image was automated (Image > Adjust > Threshold), and the automated threshold was changed into a binary image (Process > Binary >Make Binary) (**Figure 1**). The total and damaged leaf area were measured in pixels following the prompt ImageJ procedure, and the damage percentage was calculated.

### 2.6 Data analysis

All the data, including the oviposition preference, survival, pupation, and adult emergence percentage, were checked for homogeneity of variance (Bartlett test) and normality of errors (Shapiro–Wilk test). The oviposition preference, neonate larvae that mined into leaves, and adult emergence percentage data were subjected to analysis of variance (one-way ANOVA; PROC ANOVA; SAS System 2002). The mean separation was done using the Student Newman-Keuls test (SNK).

The larval growth index, standardized insect-growth index, and fitness index of *P. absoluta* on the tested host plants were calculated using the formulae (17):


$$\text{Larval growth index}=\frac{\text{Pupation }\%}{\text{Larval period}\left(\text{days}\right)}$$

$$\text{Standard growth index}=\frac{\text{Pupal weight}\left(\text{mg}\right)}{\text{Larval period}\left(\text{days}\right)}$$

$$\text{Fitness index}=\frac{\left(\text{Pupation }\%\times \text{Pupation weight }(\text{mg})\right)}{\text{Larval period }(\text{days})\times \text{Pupal period }(\text{days}))}$$

## 3 Results

### 3.1 Oviposition preference of tomato leafminer

**Figure 2:**
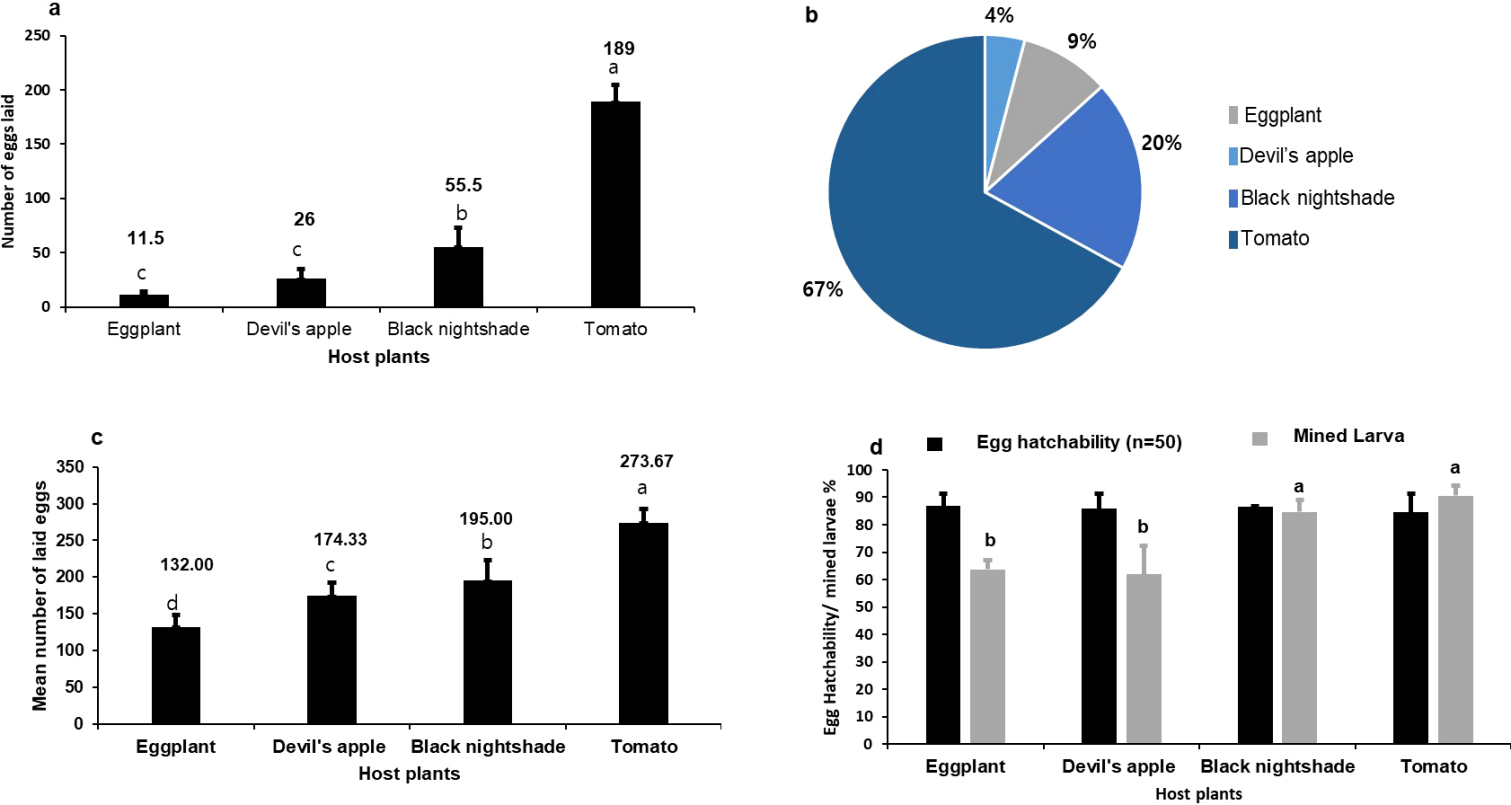
Ovipositional preference of *P. absoluta* among the Solanaceous crops and weed species. **(a)** Choice test of ovipositional preference (n=5 pairs), **(b)** Ovipositional preference % coverage; the ovipositional percentage on each plant was calculated from the total eggs laid in the cage during the choice test. **(c)** No choice test ovipositional preference (n=2 pairs); individual host plant was separately provided for oviposition. **(d)** Eggs hatchability and mined neonates %; the mined larvae were assessed after 24h of hatch. Bars followed by different letters were significantly different according to the Student Newman-Keuls test (SNK) (p< 0.05).

Females laid eggs on all four solanaceous plants, and the number of eggs laid varied significantly across host plants. The highest number of eggs was laid on the tomato plant, followed by black nightshade, whereas significantly lower numbers of eggs were laid on eggplant in both choice and no-choice tests (**Figures 2a, c**). From the mean total eggs laid under the choice test, 67% of the eggs laid were recorded from tomato plants, whereas only 4% were from eggplants (**Figure 2b**).

More than 80% of the eggs were successfully hatched across all the plants; however, the mining by the neonates was different (**Figure 2d**). Significantly higher percentages of neonates mined the tomato (90.6%) and black nightshade (84.6%) after 24 h of hatch.

### 3.2 Larval survival and mortality across instars

**Figure 3:**
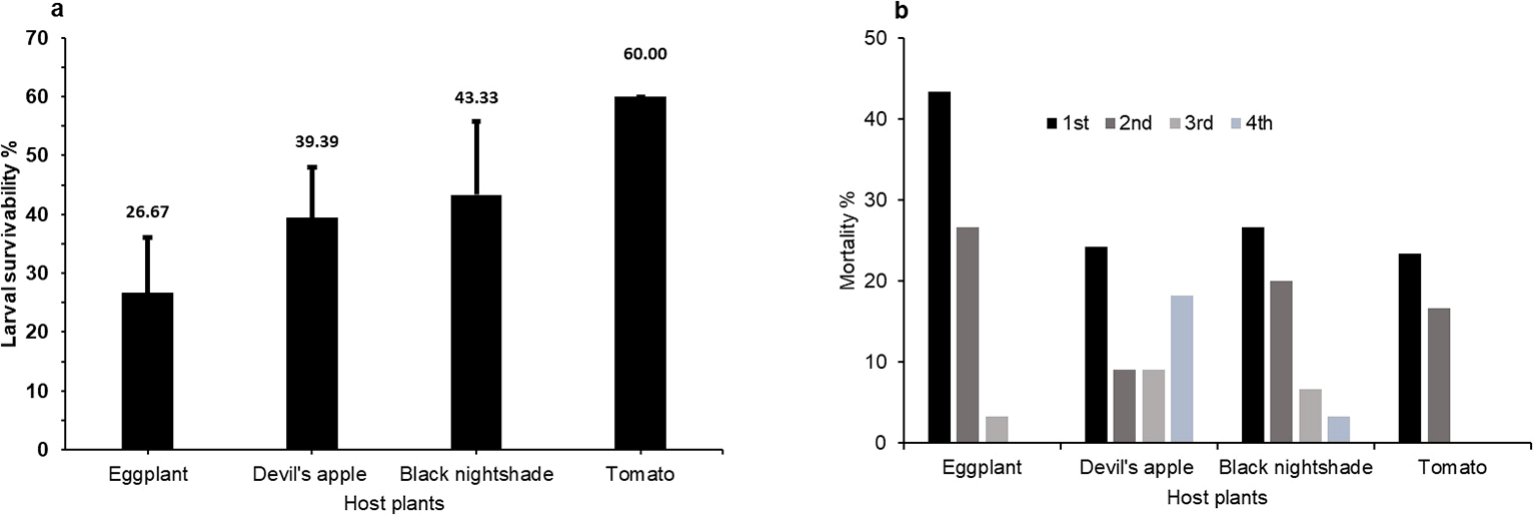
Larval fitness on different host plants. **(a)** Larval survivability percentage (n=30), **(b)** Larval mortality across the larval instars. The change of larval instars was quantified based on daily monitoring of the head capsule. Concurrently, the larval mortality at each larval instar was recorded on each tested plant.

*P. absoluta* completed its life cycle on all the tested host plants; however, the survival rate was different across the tested host plants. The survival rate of 60, 43.3, 39.4, and 26.7% was observed on tomato, black nightshade, devil’s apple, and eggplants, respectively (**Figure 3a**). The highest larval mortality was observed at the 1^st^ instar larvae on all the host plants (**Figure 3b**); however, significantly higher mortality was from eggplant, both at 1^st^ (43.3%) and 2^nd^ (26.7%) larval instars. Most of the neonate larvae died within 24 h of mining the eggplant leaf. A continuous mortality was recorded on devil’s apple and black nightshade across all the larval instars, whereas no larval mortality was observed on tomato-fed larvae after the 2^nd^ instar.

### 3.3 Larval developmental time on solanaceous plants

**Figure 4:**
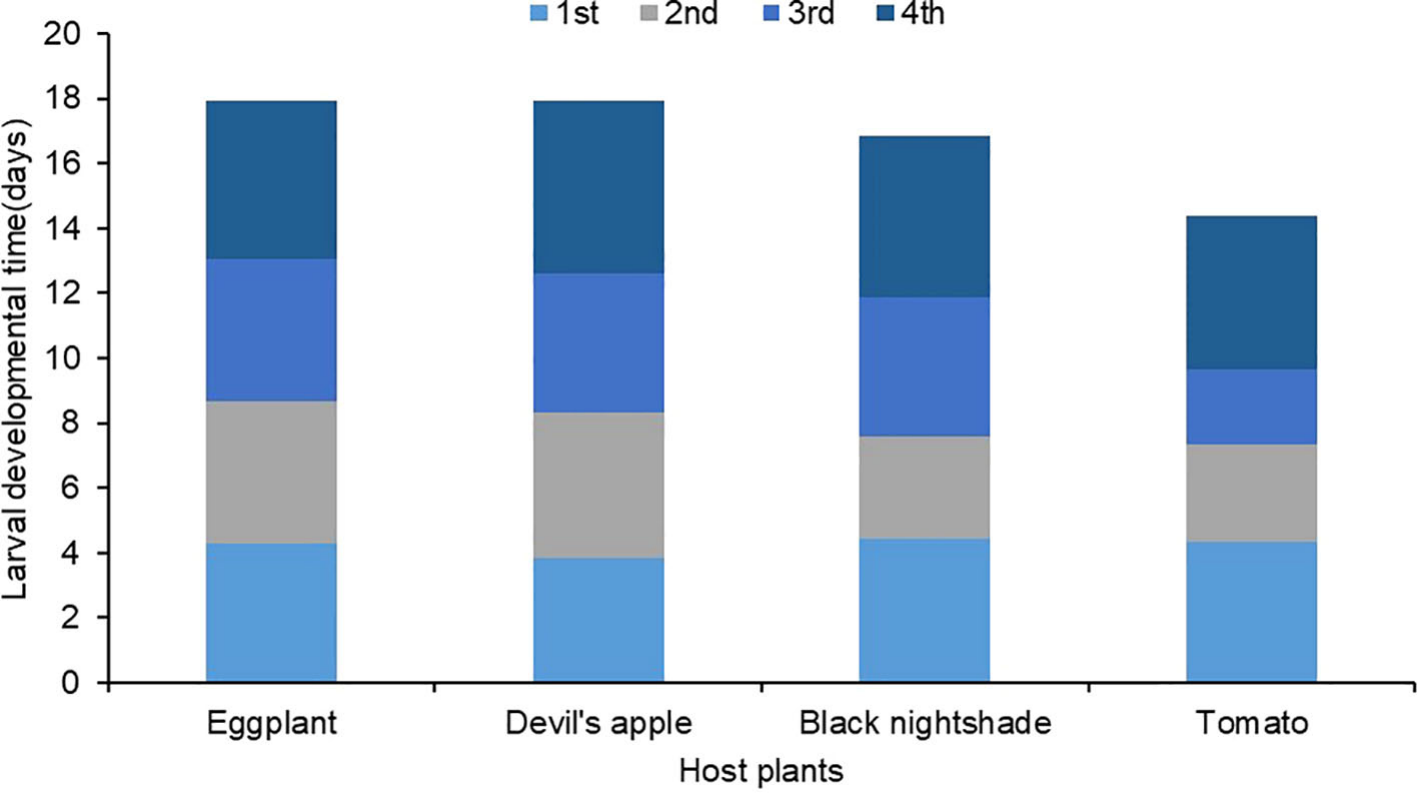
Effect of host plants on *P. asoluta* larval sdevelopment. Each larval instar was examined by daily monitoring of the head capsules.

The larval duration was longer on eggplant (18 days) and devil’s apple (18 days), and relatively shorter on black nightshade (16 days) and tomato (14 days) (**Figure 4**). While no significant difference in duration was observed among the plants during the 1^st^ instar, larvae reared on tomato plants exhibited significantly shorter developmental times during the 2^nd^ and 3^rd^ instars.

**Figure 5:**
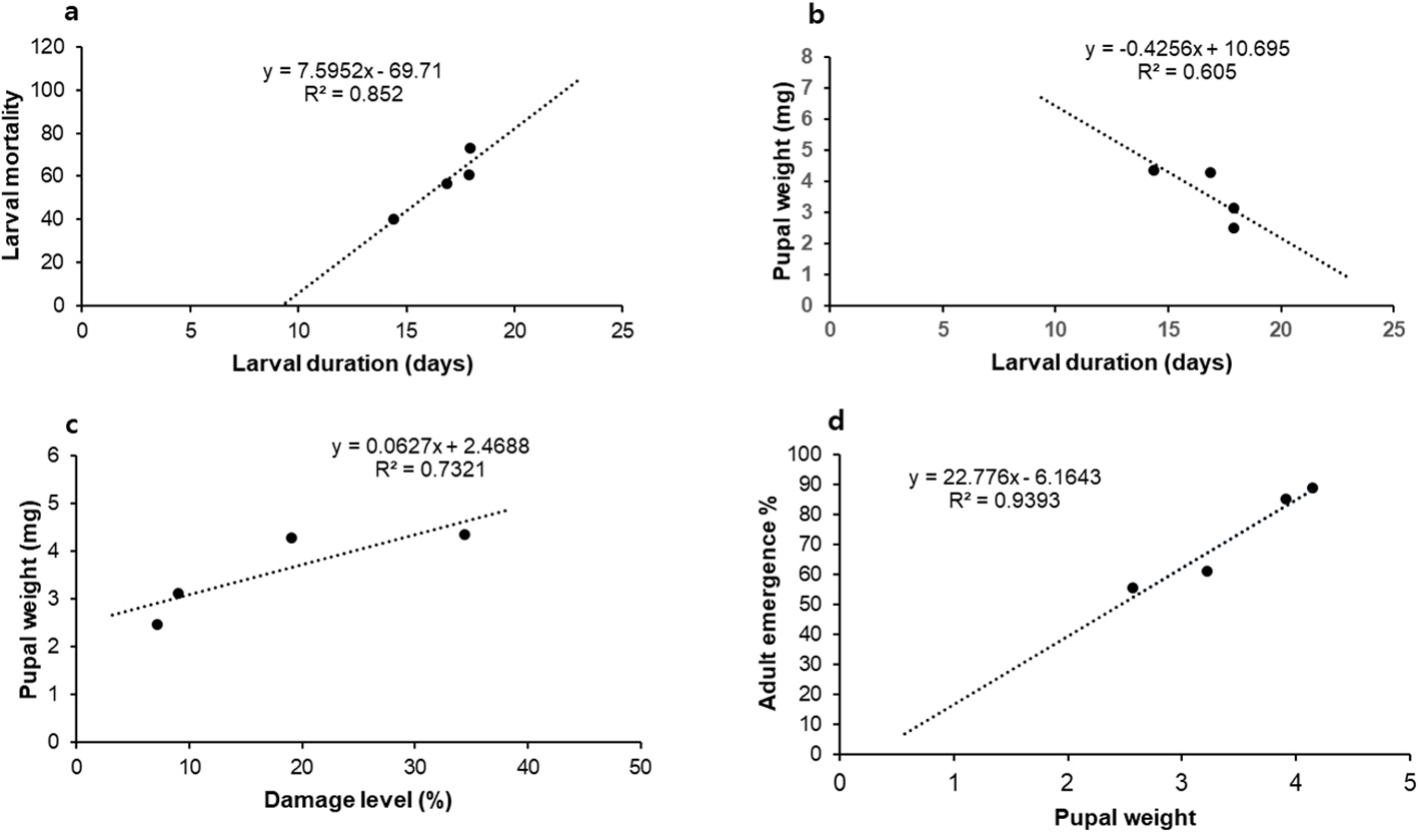
Correlation between larval fitness traits. **(a)** larval duration and larval mortality, **(b)** larval duration and pupal weight, **(c)** damage percentage and pupal weight, **(d)** pupal weight and adult emergence.

Larval duration was positively correlated with larval mortality (R² = 0.85), suggesting that longer development may be associated with increased mortality (**Figure 5a**). Moreover, the effect of larval duration on the resulting pupal weight was also analyzed, and a negative correlation was observed (R^2^ = 0.61) (**Figure 5b**). The increase in larval duration by 1-day decreases the pupal weight by 0.43mg.

### 3.4 Effect of host plants on larval fitness and growth indexes

The larval group developed on different host plant species was evaluated for fitness. The pupae were sexed and separately weighed. On all tested plant species, the weight of female pupae was higher than that of male pupae; however, the larvae developed on tomato and black nightshade resulted in significantly bigger pupae in both sexes (**Table 1**). On the contrary, the larvae developed feeding on devil’s apple measured the lowest pupal weight.

**Table 1:** Effect of host plants on larval fitness.

Host plants	Pupal weight (mg)		Adult emergence %	Sex ratio (F:M)
	Female	Male		
Eggplant	3.15	2.63	61.11 ± 7.86^b^	0.67
Devil’s apple	2.70	2.45	55.56 ± 11.33^b^	0.60
Black nightshade	4.29	3.78	85 ± 10.80^a^	1.20
Tomato	4.14	3.78	88.89 ± 7.86^a^	1.29

Means followed by different letters were significantly different according to the Student Newman-Keuls test (SNK) (p< 0.05).

The eclosion percentage revealed significant differences in adult emergence among the tested host plants (**Table 1**). Specifically, tomato and black nightshade resulted in the significantly highest eclosion percentage, whereas the lowest percentage (55.56%) was recorded for larvae reared on devil’s apple. Furthermore, sex ratio analysis indicated that a higher proportion of females emerged from larvae fed on tomato and black nightshade. In contrast, males predominated in populations developed on devil’s apple and eggplant (**Table 1**).

The growth indexes (larval, standardized, and fitness growth) of *P. absoluta* were evaluated across various host plants. The highest growth indexes were recorded on tomato (4.17, 0.29, and 4.32, respectively), followed by black nightshade (2.57, 0.23, and 1.83). Comparatively lower growth indexes were recovered on eggplant and devil’s apple (**Table 2**). Furthermore, regression analysis revealed that there is a strong positive linear correlation between pupal weight and adult emergence (R^2^ = 0.94; **Figure 5d**). Based on the regression model, adult emergence is predicted to be zero for pupae weighing less than 0.3 mg, while every 1 mg increase in weight corresponds to a 22.77% increase in emergence rate.

**Table 2:** Larval and standardized growth and fitness indices of *P. absoluta* across the host plants .

Host plants	Larval growth indices	Standardized growth indices	Fitness indices
Eggplant	1.49	0.18	0.80
Devil’s apple	2.20	0.14	0.94
Black nightshade	2.57	0.23	1.83
Tomato	4.17	0.29	4.32

### 3.5 Leaf damage analysis

**Figure 6:**
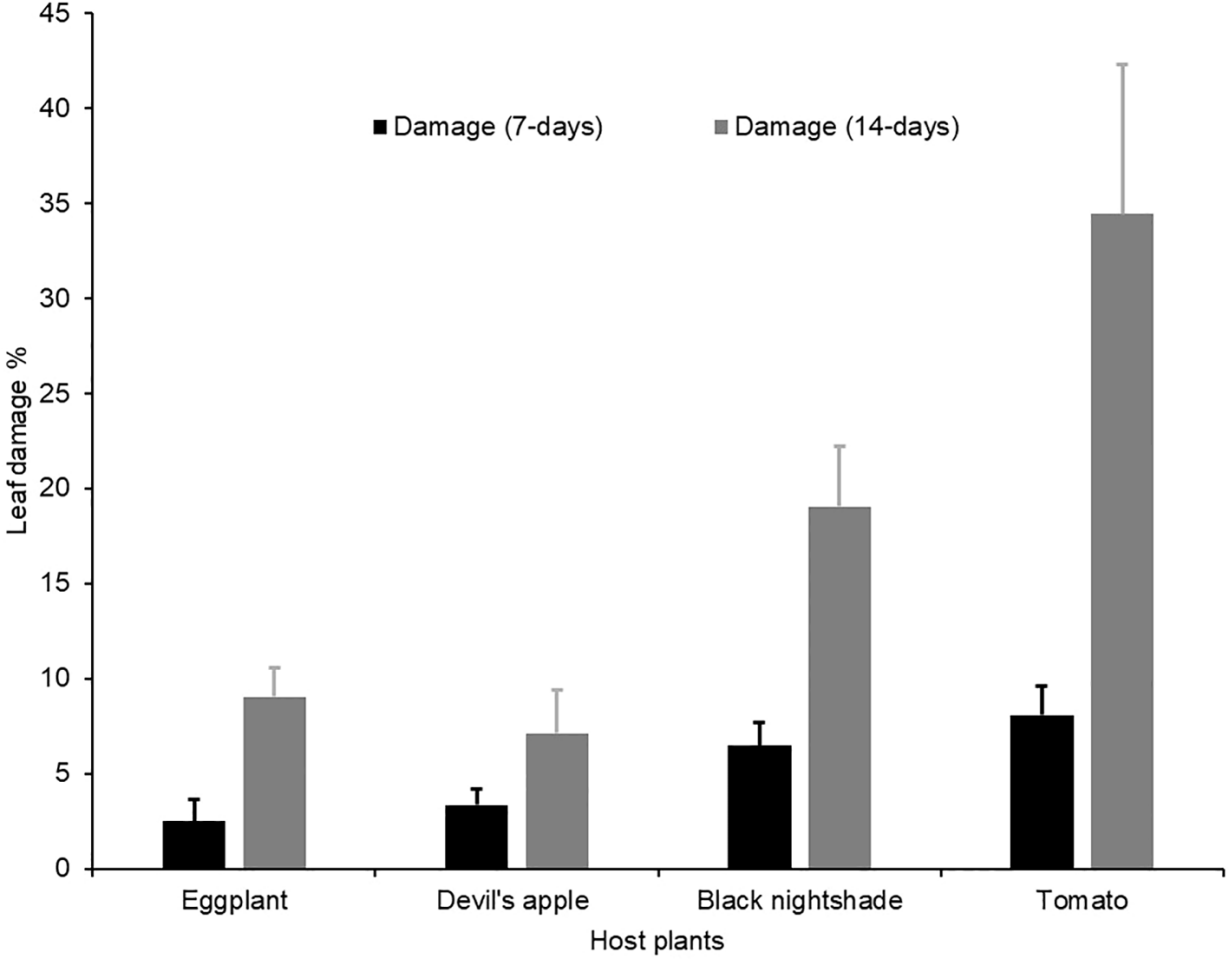
Leaf damage percentage. The damage percentage was calculated twice on the 7th and 14th days.

To understand the larval feeding potential across different host plants, the leaf damage caused by the larvae was measured twice at 7 and 14 days post-infestation (**Figure 6**). The results revealed that the highest leaf damage at 7 days post-infestation (8.06% and 6.47%) was recorded on tomato and black nightshade, respectively, whereas damage on eggplant (2.49%) and devil’s apple (3.34%) was comparatively lower. The result remained consistent after 14 days of infestation. A positive correlation was observed between damage level and pupal weight (R^2^ = 0.79), indicating that a 1% increase in damage corresponds to a 0.0627mg increase in the pupal weight (**Figure 5c**).

## 4 Discussion

Understanding the impact of the host plant on biological parameters and population growth of herbivorous insect pests is important for developing the integrated pest management strategies, especially for newly invaded species (18). We have evaluated four solanaceous plant species, such as eggplant, devil’s apple, black nightshade, and tomato, for their suitability for the development and growth of *P. absoluta*. These host plant species are commonly found in all areas of the Republic of Korea.

Accordingly, *P. absoluta* adults highly preferred tomato, followed by black nightshade, among all the plant species provided. In contrast, few eggs were laid on the eggplant and devil’s apple during both the choice and no-choice oviposition preference tests. Comparatively, more eggs were recorded during the no-choice test, indicating that, despite variations in preference levels, adults can lay eggs on other host plants in the absence of their primary host, tomato. This demonstrates that these plants could serve as potential alternative hosts for the insect’s development. The primary ovipositional host preference factors for *P. absoluta* result from evolutionary adaptation and the ability of females to choose the most favorable environment for offspring survival (15). Furthermore, volatile compounds from host plants play an essential role in discerning the appropriate host for oviposition (19). Among the identified compounds, 1-nonanol and ethyl octanoate, identified from tomato, were shown to increase the number of eggs laid by *P. absoluta*. Conversely, trans, trans-2,4-nonadienal and trans-2-nonenal (abundant in eggplants) exhibited a repellent effect on the oviposition choices of *P. absoluta* females (19). Therefore, volatile compounds released by different plants serve as semiochemicals that either attract or deter females during oviposition site selection (20).

The percentage of neonate mining was examined to assess the fitness of early instar larvae on different host plants. A significantly lower percentage of neonates successfully mined into eggplants and devil’s apple compared to tomato and black nightshade. This suggests that the physical traits of host plant leaves significantly influence both ovipositional preference and larval development. Tomato plants are characterized by a high density of type IV and VI glandular trichomes, which release attractive volatile compounds and create a favorable microenvironment for eggs and larvae (21, 22). Furthermore, tomato plants possess thin, soft, pliable, and velvety leaf surfaces (23) that provide palatable tissue and optimal nutrition for larval growth. In contrast, eggplants possess less dense and non-granular trichomes that are characterized by thicker, pubescent surfaces (24). Similarly, devil’s apple features tougher and pricklier leaf structures (25). These morphological defenses likely contribute to reducing adult oviposition preference and serve as physical barriers that delay larval development or increase mortality.

The larval survivability was evaluated to determine host plant suitability and larval performance across the selected solanaceous species. In this study, *P. absoluta* successfully completed its life cycle on all tested host plants; however, survival rates varied significantly between the host plants. The highest pupation rate was observed on tomato plants, while the lowest survivability was recorded on eggplant and devil’s apple. This trend aligns with the result of oviposition preference and neonate larvae tunneling examination. Furthermore, high mortality was especially noted during the 1^st^ instar stage on both eggplant and devil’s apple. As mentioned previously, the inability of neonates to successfully tunnel, along with high mortality observed at this stage, may be attributed to leaf texture and host plant nutritional content. These findings are consistent with Idriss et al. (26), who reported variations in *P. absoluta* larval survival among *S. lycopersicum*, *S. nigrum*, and *P. vulgaris*, with the highest larval fitness observed on *S. lycopersicum* and *S. nigrum*. In addition, several studies have demonstrated that larval survival variability across different host plants is driven by leaf texture, morphological structure, the presence of deterrent compounds, and overall nutritional quality (6, 27, 28). Consistently, the larvae reared on eggplants and devil’s apple exhibited a prolonged duration before pupation, whereas a significantly shorter larval period and higher pupation rate were observed on tomato and black nightshade. Analysis of instar-specific development revealed that the duration of the 1^st^ instar was relatively uniform across host plants; however, subsequent instars showed significantly prolonged development when fed on eggplant and devil’s apple. Insects feeding on alternative or nutritionally deficient hosts typically exhibit slower growth rates (29). Furthermore, assessments of larval feeding damage revealed that consumption rates were remarkably lower on eggplant and devil’s apple. Specifically, the larvae feeding on tomato leaves consumed 3.2-fold and 2.4-fold more tissues than those on eggplant and devil’s apple, respectively, highlighting distinct larval preferences among the tested hosts. The strong positive correlations observed between larval duration and mortality, as well as between damage levels, pupal weight, and adult emergence, suggest that the host plant unsuitability or low nutritional quality reduces feeding rates. This suppressed consumption prolongs larval development, which in turn increases mortality. Conversely, high damage levels serve as an indication of larval fitness, resulting in greater pupal weight and a higher percentage of adult emergence. Low rate of larval survival, reduced damage percentages, and long development times typically indicate poor nutritional quality in host plants (27, 30–32). In this study, the variations in duration difference between early and later instars on the tested host plants may be attributed to fluctuating nutrition demands and metabolic requirements throughout larval development. Offor (33) supports this by highlighting stage-specific requirements, metabolic constraints, and the developmental consequences of nutrient limitation. Similar performance trends have been observed in other lepidopterans; for instance, *Helicoverpa armigera* exhibits rapid development and high survivability on its primary host, chickpea, compared to slower development and higher mortality on maize (34). Furthermore, studies on *Spodoptera frugiperda* fitness traits indicate that larval duration is positively correlated with mortality and negatively correlated with pupal weight (35). The survival rates and development time of *P. absoluta* on various solanaceous species likely reflect the insect’s evolutionary adaptation and host specialization history. Consequently, the extended development period and lower larval survival rates observed on eggplants and devil’s apple suggest these plants possess inferior nutritional quality compared to tomato and black nightshade. These findings align with Coqueret et al. (36), who reported that nitrogen-limited tomato plants significantly retard *P. absoluta* development, likely due to enhanced plant defenses and lower nutritive value.

Furthermore, larval fitness traits, including pupal weight, adult emergence, and pupal duration, showed significant variation across the evaluated host plants. The lowest pupal weight was recorded in larvae reared on devil’s apple and eggplant. Similarly, growth index parameters (larval, standardized, and fitness growth indices) indicated that the development of *P. absoluta* was heavily influenced by the host plant species. The results demonstrate that *P. absoluta* exhibits optimal growth on tomato and black nightshade, whereas the reduced larval growth observed on other hosts suggests lower nutritional value or the presence of defensive metabolites. These findings align with Mousavi et al. (37), who observed that *Spodoptera littoralis* pre-pupal and pupal weights were significantly higher on coriander than on alternative hosts such as basil, dill, chives, mint, parsley, and purslane. Additionally, a strong positive correlation was observed between pupal weight and adult emergence. Likewise, observations in other lepidopteran species, such as *S. frugiperda*, show that larval duration and pupal weight vary by host plant, with pupal weight positively correlating with adult fecundity (35). Collectively, these correlations suggest that adult reproductive success is a direct consequence of larval fitness and nutritional intake during the feeding stage.

## 5 Conclusion

The tomato leafminer feeds on a wide range of host plants within the Solanaceae family. Ovipositional preference and larval fitness are critical parameters assessed to determine host suitability. While *P. absoluta* can complete its life cycle on various host plant species, larval mortality was highest on eggplants and devil’s apple, suggesting consideration of these hosts as alternate hosts in developing management strategies. Further research on host plant–pest interactions, including studies on plant physical and chemical traits, is essential to understand the host range and develop effective, sustainable pest management strategies.

## Data Availability

The raw data supporting the conclusions of this article will be made available by the authors, without undue reservation.
